# *Journal of Neurodevelopmental Disorders *is now a fully open access journal

**DOI:** 10.1186/1866-1955-4-1

**Published:** 2012-02-08

**Authors:** Joseph Piven

**Affiliations:** 1 Carolina Institute for Developmental Disabilities, University of North Carolina at Chapel Hill, Chapel Hill, NC 27599-3366, USA

## Editorial

With the publication of the first articles of this 2012 volume, the *Journal of Neurodevelopmental Disorders *enters the exciting world of open access publishing. The Journal will now be published by BioMed Central, (part of Springer Science+Business Media) as a fully open access journal. This change will mean that all articles will be made freely and permanently accessible online immediately upon publication, will be available to readers throughout the world without subscription charges or registration barriers, and be indexed in both PubMed and PubMed Central.

In line with BioMed Central's copyright and license policy, authors will retain the copyright to their work and grant anyone the right to reproduce and disseminate their articles, as long as the integrity of the original work is maintained and it is correctly cited (http://www.biomedcentral.com/about/copyright). Authors submitting from BioMed Central Member institutions have article processing charges covered in full or in part by their institutions (http://www.biomedcentral.com/libraries/membership) and authors from countries in low or low-middle income categories (as defined by the World Bank) may have charges waived (http://www.biomedcentral.com/authors/oawaiverfund). Other waivers may be available on a case by case basis.

Given the high relevance of the work published in this journal to families, clinicians and researchers throughout the world, we are committed to this new and equitable approach to dissemination of scientific findings in our field.

The Journal provides a unique forum for the integration of knowledge across a number of neurodevelopmental disorders, underlying pathogenetic mechanisms, and scientific disciplines relevant to understanding the pathogenesis of neurodevelopmental disorders.

In the Foreward to the inaugural issue of the Journal, Tom Insel, Director of the National Institutes of Mental Health, wrote "There has never been a better time for this integration and never a greater need" [1]. The most recent issue (vol 3, issue 4) [2] included papers on Turner Syndrome, Autism Spectrum Disorder, Prader-Willi Syndrome, William's Syndrome, Fragile X, Specific Language Impairment and Stuttering, and covered a wide-range of cross-cutting perspectives from the effects of prenatal exposures on risk for autism to the impact of sex differences on the developing brain. In this same issue, the Special Section entitled 'Building an Epigenetics Perspective on Language, Speech and Reading Impairments', edited by Mabel Rice [3], examined in more detail genetic mechanisms underlying disorders of communication. Clearly this new Journal has met the challenge of integrating across multiple perspectives and over multiple levels of analysis.

The latest research in the field suggests the need to go beyond contrasting disease states with typical development, instead comparing and contrasting the overlapping phenomenology across conditions and asking such questions as - What are the commonalities and differences in social cognition between autism and schizophrenia [4]?; and What is the unique brain morphology in children with autism associated with the presence of the Fragile X mutation versus the brain as seen in those with autistic behavior without an identifiable, associated etiologic factor [5]? With the same rare mutations being found in association with multiple phenotypes (e.g., global developmental delay, in the case of copy number variation of the 16p11 region), there is a critical need for studies which cut across traditional phenotypic boundaries linked to the most salient aspects of clinical impairment. While traditional clinical phenotypes are still of primary importance to clinicians, research findings are pointing more towards the need to look beyond these classical nosologies for alternate conceptualizations that may better map on to components of common, converging molecular pathways, or neural systems, with notions about risk and susceptibility better reflecting underlying pathogenetic mechanisms than classifications relying entirely on clinically-derived definitions. In the area of intervention, targeted approaches to treatment now require us to integrate across disciplines and bioinformatics domains. So, for example, the recent discovery of the ability of drugs used in the treatment of pediatric cancers to unsilence the Ube3a gene in a mouse model of Angelman Syndrome and autism, points to the urgent need for integration across disciplines and levels of inquiry [6].

These are truly exciting times for scientists working to unravel the basis of neurodevelopmental disorders. The tools we have at hand for both the discovery and dissemination of new knowledge are unprecedented. By bringing together multiple perspectives on the pathogenesis of neurodevelopmental disorders in this new Journal, together with cutting-edge approaches for distributing this new knowledge throughout our scientific community, the *Journal of Neurodevelopmental Disorders *is poised to make an important contribution to our field. We hope you share our enthusiasm for these advances. We encourage you to consider submission of your work to the Journal.
